# Crystal structure of (*Z*)-ethyl 3-[2-(5-methyl-7-nitro-1*H*-indole-2-carbon­yl)hydrazinyl­idene]butano­ate

**DOI:** 10.1107/S2056989015015054

**Published:** 2015-08-22

**Authors:** Amal Errossafi, Abdellatif El Kihel, Salaheddine Guesmi, Mohamed Saadi, Lahcen El Ammari

**Affiliations:** a Laboratoire de chimie bioorganique, Faculté des Sciences, Université Chouaib Doukkali, BP 20, M-24000 El Jadida, Morocco; b Laboratoire de Chimie de Coordination et d’Analytique (LCCA), Faculté des Sciences, Université Chouaib Doukkali, BP 20, M-24000 El Jadida, Morocco; c Laboratoire de Chimie du Solide Appliquée, Faculté des Sciences, Université Mohammed V, Avenue Ibn Battouta, BP 1014, Rabat, Morocco

**Keywords:** crystal structure, conformation, hydrogen bonding

## Abstract

The reaction of 5-methyl-7-nitro-1*H*-indole-2-carbohydrazide with ethyl aceto­acetate yielded the title mol­ecule, C_16_H_18_N_4_O_5_, in which the indole ring is almost planar, with the greatest deviation from the mean plane being 0.006 (2) Å. The nine atoms of the indole ring are almost perpendicular to the mean plane through the ethyl acetate group, as indicated by the dihedral angle of 87.02 (4)° between them. In the crystal, centrosymmetric supra­molecular dimers are formed *via* N—H⋯O hydrogen bonds and eight-membered amide {⋯HNCO}_2_ synthons. These are consolidated into a three-dimensional architecture by C—H⋯O contacts, and by π–π inter­actions between six-membered rings [inter-centroid distance = 3.499 (2) Å].

## Related literature   

For biochemical properties of indoles, see: Kuethe *et al.* (2005 ▸); Smith *et al.* (1998 ▸). For medicinal activity, see: El Kihel *et al.* (2007 ▸, 2013 ▸); Penning *et al.* (1997 ▸); Dumas *et al.* (2000 ▸). For starting materials, see: El Ouar *et al.* (1995 ▸).
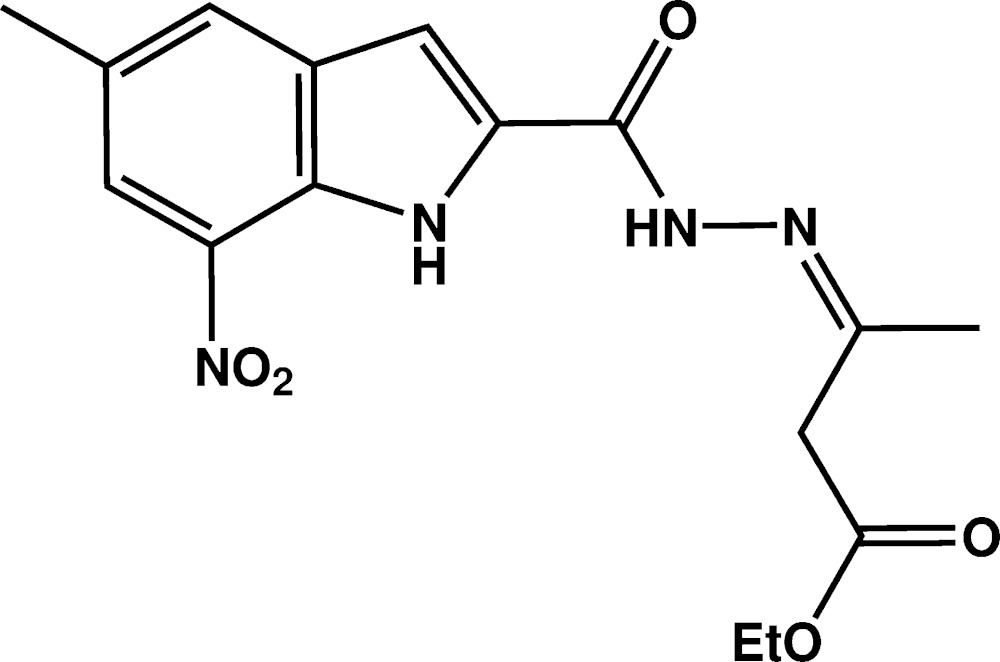


## Experimental   

### Crystal data  

C_16_H_18_N_4_O_5_
*M* *_r_* = 346.34
Triclinic, 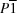

*a* = 8.4716 (9) Å
*b* = 8.4722 (7) Å
*c* = 13.0971 (9) Å
α = 108.695 (4)°
β = 91.865 (4)°
γ = 106.886 (4)°
*V* = 843.80 (13) Å^3^
*Z* = 2
Mo *K*α radiation
μ = 0.10 mm^−1^
*T* = 296 K
0.35 × 0.30 × 0.27 mm

### Data collection  

Bruker X8 APEX diffractometer
Absorption correction: multi-scan (*SADABS*; Bruker, 2009 ▸) *T*_min_ = 0.589, *T*_max_ = 0.746
18286 measured reflections
3676 independent reflections
3120 reflections with *I* > 2σ(*I*)
*R*_int_ = 0.033

### Refinement  

*R*[*F* ^2^ > 2σ(*F* ^2^)] = 0.043
*wR*(*F* ^2^) = 0.124
*S* = 1.07
3676 reflections
226 parameters
H-atom parameters constrained
Δρ_max_ = 0.30 e Å^−3^
Δρ_min_ = −0.24 e Å^−3^

### 

Data collection: *APEX2* (Bruker, 2009 ▸); cell refinement: *SAINT* (Bruker, 2009 ▸); data reduction: *SAINT*; program(s) used to solve structure: *SHELXS97* (Sheldrick, 2008 ▸); program(s) used to refine structure: *SHELXL97* (Sheldrick, 2008 ▸); molecular graphics: *ORTEP-3 for Windows* (Farrugia, 2012 ▸); software used to prepare material for publication: *PLATON* (Spek, 2009 ▸) and *publCIF* (Westrip, 2010 ▸).

## Supplementary Material

Crystal structure: contains datablock(s) I. DOI: 10.1107/S2056989015015054/tk5378sup1.cif

Structure factors: contains datablock(s) I. DOI: 10.1107/S2056989015015054/tk5378Isup2.hkl

Supporting information file. DOI: 10.1107/S2056989015015054/tk5378Isup3.cml

**Fig. 1.**
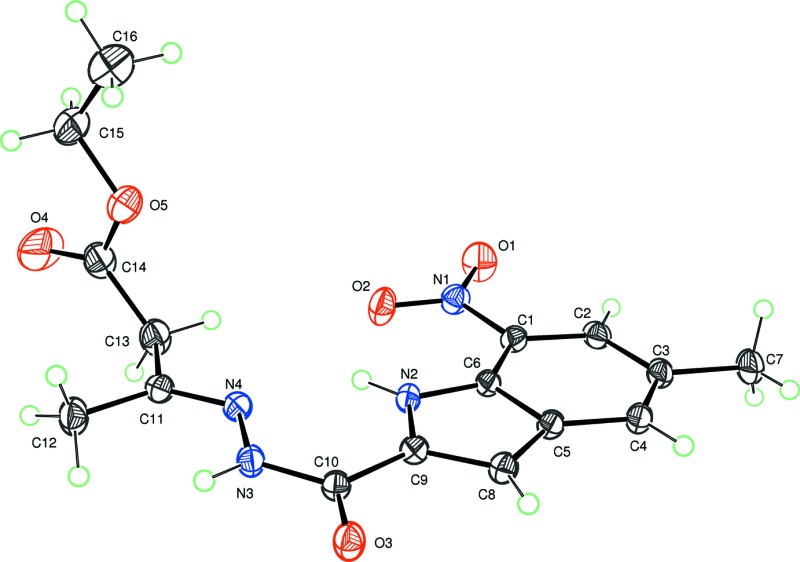
Plot of the molecule with the atom-labelling scheme. Displacement ellipsoids are drawn at the 50% probability level.

. DOI: 10.1107/S2056989015015054/tk5378fig1.tif
Plot of the mol­ecule with the atom-labelling scheme. Displacement ellipsoids are drawn at the 50% probability level.

**Fig. 2.**
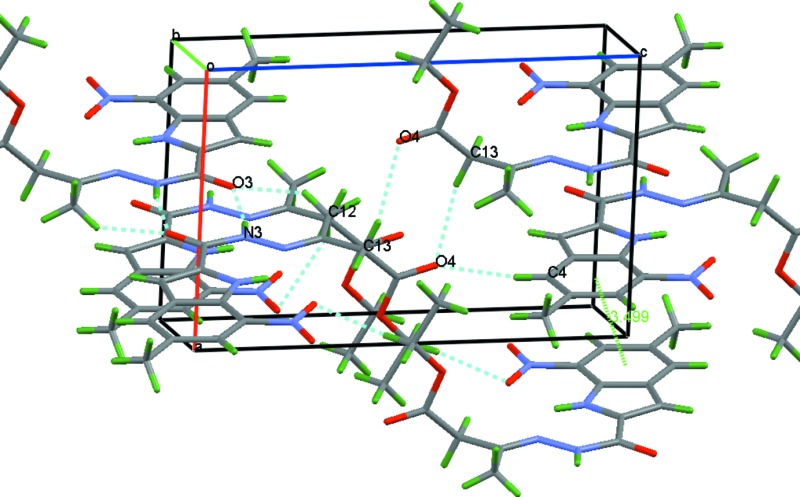
A view of the crystal packing of the title compound, showing intermolecular π—π interactions between six-membered rings (dashed green lines) and intermolecular hydrogen bonds (dashed blue lines).

. DOI: 10.1107/S2056989015015054/tk5378fig2.tif
A view of the crystal packing of the title compound, showing inter­molecular π—π inter­actions between six-membered rings (dashed green lines) and inter­molecular hydrogen bonds (dashed blue lines).

CCDC reference: 1418363

Additional supporting information:  crystallographic information; 3D view; checkCIF report

## Figures and Tables

**Table 1:** Hydrogen-bond geometry (, )

*D*H*A*	*D*H	H*A*	*D* *A*	*D*H*A*
N3H3*N*O3^i^	0.86	2.04	2.8815(15)	167
C12H12*C*O3^i^	0.96	2.39	3.1723(18)	139
C4H4O4^ii^	0.93	2.51	3.4019(19)	161
C13H13*A*O4^iii^	0.97	2.56	3.407(2)	146

Symmetry codes: (i) 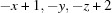; (ii) 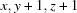; (iii) 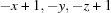.

## supplementary crystallographic information

### S1. Comment

The indole nucleus is probably the most widely distributed heterocyclic ring system found in nature (Kuethe *et al.*, 2005). Due to the existence of a vast array of structurally diverse and biologically active indoles, it is not surprising that the indole nucleus is an important feature in many medicinal agents and the most important of all structural classes in drug discovery (Smith *et al.*, 1998). For example, the identification of new and selective cox-2 inhibitors (Penning *et al.*, 1997), for the relief of pain, and the treatment of the symptoms of arthritis and related diseases has been an important advance in modern anti-inflammatory therapy. In a related area, heterocycle-appended pyrazoles have been reported (Dumas *et al.*, 2000) to be potent and selective as inhibitors of the mitogen-activated protein kinase p38 and consequently provide a novel approach for the treatment of rheumatoid arthritis and related inflammatory diseases. The synthesis and reactivity of indole derivatives have been a topic of research interest for well over a century. Due to the potent biological activity exhibited by various indoles derivatives, there is a continuous demand for novel synthetic procedures in this area. In previous papers (El Kihel *et al.*, 2007; 2013; El Ouar *et al.*, 1995), we have reported some reactions of 7-aminoindoles and the condensation of the 7-nitroindole-2-carbohydrazide with acetylacetone. Herein, we report the synthesis of an open intermediate, 5-methyl-7-nitroindole-2-carbohydrazide, by condensation with ethyl acetoacetate.

The fused five- and six-membered rings, part of the title compound, are essentially planar with the largest deviation from the mean plane being 0.006 (2) Å at C1 atom as shown in Fig. 1. The mean plane through the ethyl acetate group is virtually perpendicular to the indasol ring as indicated by the dihedral angle of 87.02 (4) ° between them. The cohesion of the crystal is ensured by C—H···O and N—H···O hydrogen bonds, and by π—π interactions between phenyl rings [intercentroid distance = 3.499 (2) Å], as shown in Fig. 2 and Table 2.

### S2. Experimental

The ethyl 3-(2-(5-methyl-7-nitro-1*H*-indole-2-cabonyl)hydrazono)butanoate was synthesized from a mixture of 5-methyl-7-nitroindole-2-carbohydrazide (4.6 mmol) and ethyl acetoacetate (35 mmol) in ethanol which was heated on a steam bath at 353 K until dissolution. The mixture was kept at this temperature for 4 h. The crude product was filtered and crystallized from ethanol. Yellow crystals appeared after two weeks. The crystals were washed with cold ethanol and dried in air at room temperature.

### S3. Refinement

The H atoms were located in a difference map and treated as riding with C—H = 0.93 Å (aromatic), C—H = 0.97 Å (methylene) and C—H = 0.96 Å (methyl), and with *U*_iso_(H) = 1.2 *U*_eq_ (aromatic and methylene) and *U*_iso_(H) = 1.5 *U*_eq_ (methyl).

### Crystal data

**Table:**

C_16_H_18_N_4_O_5_	*Z* = 2
*M**_r_* = 346.34	*F*(000) = 364
Triclinic, *P*1	*D*_x_ = 1.363 Mg m^−^^3^
*a* = 8.4716 (9) Å	Mo *K*α radiation, λ = 0.71073 Å
*b* = 8.4722 (7) Å	Cell parameters from 3676 reflections
*c* = 13.0971 (9) Å	θ = 2.5–27.1°
α = 108.695 (4)°	µ = 0.10 mm^−^^1^
β = 91.865 (4)°	*T* = 296 K
γ = 106.886 (4)°	Block, yellow
*V* = 843.80 (13) Å^3^	0.35 × 0.30 × 0.27 mm

### Data collection

**Table:**

Bruker X8 APEX diffractometer	3676 independent reflections
Radiation source: fine-focus sealed tube	3120 reflections with *I* > 2σ(*I*)
Graphite monochromator	*R*_int_ = 0.033
φ and ω scans	θ_max_ = 27.1°, θ_min_ = 2.5°
Absorption correction: multi-scan (*SADABS*; Bruker, 2009)	*h* = −10→10
*T*_min_ = 0.589, *T*_max_ = 0.746	*k* = −10→10
18286 measured reflections	*l* = −16→16

### Refinement

**Table:**

Refinement on *F*^2^	Primary atom site location: structure-invariant direct methods
Least-squares matrix: full	Secondary atom site location: difference Fourier map
*R*[*F*^2^ > 2σ(*F*^2^)] = 0.043	Hydrogen site location: inferred from neighbouring sites
*wR*(*F*^2^) = 0.124	H-atom parameters constrained
*S* = 1.07	*w* = 1/[σ^2^(*F*_o_^2^) + (0.059*P*)^2^ + 0.3197*P*] where *P* = (*F*_o_^2^ + 2*F*_c_^2^)/3
3676 reflections	(Δ/σ)_max_ < 0.001
226 parameters	Δρ_max_ = 0.30 e Å^−^^3^
0 restraints	Δρ_min_ = −0.24 e Å^−^^3^

### Special details

**Table:**

Geometry. All e.s.d.'s (except the e.s.d. in the dihedral angle between two l.s. planes) are estimated using the full covariance matrix. The cell e.s.d.'s are taken into account individually in the estimation of e.s.d.'s in distances, angles and torsion angles; correlations between e.s.d.'s in cell parameters are only used when they are defined by crystal symmetry. An approximate (isotropic) treatment of cell e.s.d.'s is used for estimating e.s.d.'s involving l.s. planes.
Refinement. Refinement of *F*^2^ against all reflections. The weighted *R*-factor *wR* and goodness of fit *S* are based on *F*^2^, conventional *R*-factors *R* are based on *F*, with *F* set to zero for negative *F*^2^. The threshold expression of *F*^2^ > σ(*F*^2^) is used only for calculating *R*-factors(gt) *etc*. and is not relevant to the choice of reflections for refinement. *R*-factors based on *F*^2^ are statistically about twice as large as those based on *F*, and *R*- factors based on all data will be even larger.

### Fractional atomic coordinates and isotropic or equivalent isotropic displacement parameters (Å^2^)

**Table:**

	*x*	*y*	*z*	*U*_iso_*/*U*_eq_	
C1	0.17198 (16)	0.64599 (17)	0.94250 (11)	0.0241 (3)	
C2	0.12573 (17)	0.78350 (18)	1.00943 (12)	0.0266 (3)	
H2	0.0884	0.8540	0.9793	0.032*	
C3	0.13423 (17)	0.81836 (18)	1.12214 (12)	0.0272 (3)	
C4	0.18973 (17)	0.71346 (18)	1.16710 (11)	0.0265 (3)	
H4	0.1948	0.7356	1.2416	0.032*	
C5	0.23851 (16)	0.57373 (17)	1.10067 (10)	0.0233 (3)	
C6	0.22965 (16)	0.54061 (16)	0.98701 (10)	0.0215 (3)	
C7	0.0821 (2)	0.9697 (2)	1.19212 (14)	0.0366 (4)	
H7A	0.1600	1.0786	1.1935	0.055*	
H7B	0.0803	0.9670	1.2648	0.055*	
H7C	−0.0270	0.9596	1.1625	0.055*	
C8	0.30024 (17)	0.44531 (18)	1.12041 (11)	0.0250 (3)	
H8	0.3193	0.4334	1.1875	0.030*	
C9	0.32627 (16)	0.34231 (17)	1.02184 (11)	0.0228 (3)	
C10	0.39564 (16)	0.19582 (17)	1.00510 (10)	0.0227 (3)	
C11	0.39038 (17)	0.03684 (19)	0.72021 (11)	0.0264 (3)	
C12	0.4537 (2)	−0.1170 (2)	0.69488 (12)	0.0343 (3)	
H12A	0.5004	−0.1320	0.6280	0.052*	
H12B	0.3634	−0.2211	0.6877	0.052*	
H12C	0.5378	−0.0967	0.7527	0.052*	
C13	0.3494 (2)	0.0920 (2)	0.62686 (12)	0.0360 (4)	
H13A	0.4517	0.1398	0.6004	0.043*	
H13B	0.2990	0.1842	0.6527	0.043*	
C14	0.2322 (2)	−0.0586 (2)	0.53418 (12)	0.0394 (4)	
C15	−0.0299 (2)	−0.2793 (3)	0.48056 (15)	0.0516 (5)	
H15A	0.0177	−0.3734	0.4529	0.062*	
H15B	−0.0586	−0.2445	0.4206	0.062*	
C16	−0.1808 (3)	−0.3393 (3)	0.5309 (2)	0.0656 (6)	
H16A	−0.2618	−0.4381	0.4773	0.098*	
H16B	−0.2271	−0.2453	0.5575	0.098*	
H16C	−0.1508	−0.3731	0.5901	0.098*	
N1	0.15863 (16)	0.61115 (16)	0.82643 (10)	0.0296 (3)	
N2	0.28297 (14)	0.40043 (14)	0.94143 (9)	0.0223 (2)	
H2N	0.2888	0.3552	0.8733	0.027*	
N3	0.41081 (15)	0.09582 (15)	0.90428 (9)	0.0247 (3)	
H3N	0.4466	0.0082	0.8968	0.030*	
N4	0.36975 (14)	0.13223 (15)	0.81339 (9)	0.0251 (3)	
O1	0.10064 (17)	0.70060 (17)	0.78835 (10)	0.0465 (3)	
O2	0.20654 (16)	0.49110 (15)	0.76987 (8)	0.0386 (3)	
O3	0.43926 (13)	0.16440 (13)	1.08520 (8)	0.0298 (2)	
O4	0.2639 (2)	−0.1084 (3)	0.44345 (11)	0.0763 (5)	
O5	0.08886 (16)	−0.13058 (17)	0.56453 (9)	0.0447 (3)	

### Atomic displacement parameters (Å^2^)

**Table:**

	*U*^11^	*U*^22^	*U*^33^	*U*^12^	*U*^13^	*U*^23^
C1	0.0223 (6)	0.0229 (6)	0.0240 (6)	0.0050 (5)	0.0025 (5)	0.0060 (5)
C2	0.0232 (7)	0.0218 (6)	0.0336 (7)	0.0075 (5)	0.0029 (5)	0.0079 (5)
C3	0.0228 (7)	0.0218 (6)	0.0312 (7)	0.0062 (5)	0.0048 (5)	0.0023 (5)
C4	0.0260 (7)	0.0256 (7)	0.0225 (6)	0.0069 (5)	0.0047 (5)	0.0023 (5)
C5	0.0216 (6)	0.0228 (6)	0.0216 (6)	0.0055 (5)	0.0030 (5)	0.0039 (5)
C6	0.0195 (6)	0.0201 (6)	0.0215 (6)	0.0053 (5)	0.0033 (5)	0.0037 (5)
C7	0.0363 (8)	0.0299 (8)	0.0398 (8)	0.0162 (6)	0.0074 (7)	0.0017 (6)
C8	0.0258 (7)	0.0269 (7)	0.0211 (6)	0.0082 (5)	0.0035 (5)	0.0067 (5)
C9	0.0213 (6)	0.0224 (6)	0.0229 (6)	0.0066 (5)	0.0026 (5)	0.0057 (5)
C10	0.0217 (6)	0.0227 (6)	0.0223 (6)	0.0067 (5)	0.0035 (5)	0.0061 (5)
C11	0.0244 (7)	0.0314 (7)	0.0218 (6)	0.0104 (6)	0.0030 (5)	0.0060 (5)
C12	0.0435 (9)	0.0382 (8)	0.0241 (7)	0.0221 (7)	0.0063 (6)	0.0061 (6)
C13	0.0445 (9)	0.0442 (9)	0.0261 (7)	0.0230 (7)	0.0069 (6)	0.0126 (6)
C14	0.0464 (10)	0.0562 (10)	0.0224 (7)	0.0294 (8)	0.0039 (6)	0.0107 (7)
C15	0.0524 (11)	0.0524 (11)	0.0374 (9)	0.0216 (9)	−0.0083 (8)	−0.0040 (8)
C16	0.0566 (13)	0.0561 (12)	0.0642 (14)	0.0105 (10)	−0.0010 (10)	0.0021 (11)
N1	0.0331 (7)	0.0295 (6)	0.0266 (6)	0.0106 (5)	0.0026 (5)	0.0098 (5)
N2	0.0246 (6)	0.0223 (5)	0.0190 (5)	0.0090 (4)	0.0039 (4)	0.0042 (4)
N3	0.0292 (6)	0.0250 (6)	0.0219 (6)	0.0137 (5)	0.0035 (5)	0.0063 (5)
N4	0.0253 (6)	0.0290 (6)	0.0208 (5)	0.0110 (5)	0.0022 (4)	0.0064 (5)
O1	0.0654 (8)	0.0529 (7)	0.0374 (6)	0.0334 (7)	0.0064 (6)	0.0237 (6)
O2	0.0557 (7)	0.0387 (6)	0.0236 (5)	0.0232 (5)	0.0058 (5)	0.0062 (5)
O3	0.0398 (6)	0.0314 (5)	0.0231 (5)	0.0184 (5)	0.0058 (4)	0.0094 (4)
O4	0.0659 (10)	0.1143 (14)	0.0251 (6)	0.0216 (9)	0.0100 (6)	−0.0011 (7)
O5	0.0476 (7)	0.0525 (7)	0.0261 (5)	0.0178 (6)	0.0013 (5)	0.0019 (5)

### Geometric parameters (Å, º)

**Table:**

C1—C2	1.3827 (19)	C11—C12	1.496 (2)
C1—C6	1.3943 (19)	C11—C13	1.505 (2)
C1—N1	1.4465 (18)	C12—H12A	0.9600
C2—C3	1.404 (2)	C12—H12B	0.9600
C2—H2	0.9300	C12—H12C	0.9600
C3—C4	1.385 (2)	C13—C14	1.510 (2)
C3—C7	1.5100 (19)	C13—H13A	0.9700
C4—C5	1.4059 (18)	C13—H13B	0.9700
C4—H4	0.9300	C14—O4	1.195 (2)
C5—C6	1.4188 (18)	C14—O5	1.327 (2)
C5—C8	1.4234 (19)	C15—O5	1.453 (2)
C6—N2	1.3585 (16)	C15—C16	1.490 (3)
C7—H7A	0.9600	C15—H15A	0.9700
C7—H7B	0.9600	C15—H15B	0.9700
C7—H7C	0.9600	C16—H16A	0.9600
C8—C9	1.3729 (18)	C16—H16B	0.9600
C8—H8	0.9300	C16—H16C	0.9600
C9—N2	1.3778 (17)	N1—O1	1.2276 (17)
C9—C10	1.4803 (19)	N1—O2	1.2347 (16)
C10—O3	1.2325 (16)	N2—H2N	0.8600
C10—N3	1.3547 (17)	N3—N4	1.3798 (16)
C11—N4	1.2793 (17)	N3—H3N	0.8600
			
C2—C1—C6	119.89 (13)	C11—C12—H12B	109.5
C2—C1—N1	119.77 (13)	H12A—C12—H12B	109.5
C6—C1—N1	120.34 (12)	C11—C12—H12C	109.5
C1—C2—C3	121.06 (13)	H12A—C12—H12C	109.5
C1—C2—H2	119.5	H12B—C12—H12C	109.5
C3—C2—H2	119.5	C11—C13—C14	112.44 (13)
C4—C3—C2	119.56 (12)	C11—C13—H13A	109.1
C4—C3—C7	121.18 (13)	C14—C13—H13A	109.1
C2—C3—C7	119.26 (14)	C11—C13—H13B	109.1
C3—C4—C5	120.34 (13)	C14—C13—H13B	109.1
C3—C4—H4	119.8	H13A—C13—H13B	107.8
C5—C4—H4	119.8	O4—C14—O5	123.36 (17)
C4—C5—C6	119.39 (13)	O4—C14—C13	124.16 (18)
C4—C5—C8	134.22 (13)	O5—C14—C13	112.47 (13)
C6—C5—C8	106.39 (11)	O5—C15—C16	107.43 (15)
N2—C6—C1	132.14 (12)	O5—C15—H15A	110.2
N2—C6—C5	108.10 (12)	C16—C15—H15A	110.2
C1—C6—C5	119.76 (12)	O5—C15—H15B	110.2
C3—C7—H7A	109.5	C16—C15—H15B	110.2
C3—C7—H7B	109.5	H15A—C15—H15B	108.5
H7A—C7—H7B	109.5	C15—C16—H16A	109.5
C3—C7—H7C	109.5	C15—C16—H16B	109.5
H7A—C7—H7C	109.5	H16A—C16—H16B	109.5
H7B—C7—H7C	109.5	C15—C16—H16C	109.5
C9—C8—C5	107.13 (12)	H16A—C16—H16C	109.5
C9—C8—H8	126.4	H16B—C16—H16C	109.5
C5—C8—H8	126.4	O1—N1—O2	122.89 (13)
C8—C9—N2	109.32 (12)	O1—N1—C1	119.29 (12)
C8—C9—C10	125.34 (12)	O2—N1—C1	117.82 (12)
N2—C9—C10	125.31 (12)	C6—N2—C9	109.07 (11)
O3—C10—N3	119.72 (12)	C6—N2—H2N	125.5
O3—C10—C9	118.74 (12)	C9—N2—H2N	125.5
N3—C10—C9	121.53 (12)	C10—N3—N4	121.12 (11)
N4—C11—C12	127.75 (13)	C10—N3—H3N	119.4
N4—C11—C13	114.81 (13)	N4—N3—H3N	119.4
C12—C11—C13	117.42 (12)	C11—N4—N3	118.81 (12)
C11—C12—H12A	109.5	C14—O5—C15	115.98 (13)

### Hydrogen-bond geometry (Å, º)

**Table:**

*D*—H···*A*	*D*—H	H···*A*	*D*···*A*	*D*—H···*A*
N3—H3*N*···O3^i^	0.86	2.04	2.8815 (15)	167
C12—H12*C*···O3^i^	0.96	2.39	3.1723 (18)	139
C4—H4···O4^ii^	0.93	2.51	3.4019 (19)	161
C13—H13*A*···O4^iii^	0.97	2.56	3.407 (2)	146

Symmetry codes: (i) −*x*+1, −*y*, −*z*+2; (ii) *x*, *y*+1, *z*+1; (iii) −*x*+1, −*y*, −*z*+1.
