# Supplementary figures and images for: Crystal structure of (Z)-ethyl 3-[2-(5-methyl-7-nitro-1H-indole-2-carbon­yl)hydrazinyl­idene]butano­ate
Source: Acta Crystallogr E Crystallogr Commun. 2015 Aug 22;71(Pt 9):o684–5. doi: 10.1107/S2056989015015054 (PMC4555398; doi:10.1107/S2056989015015054)

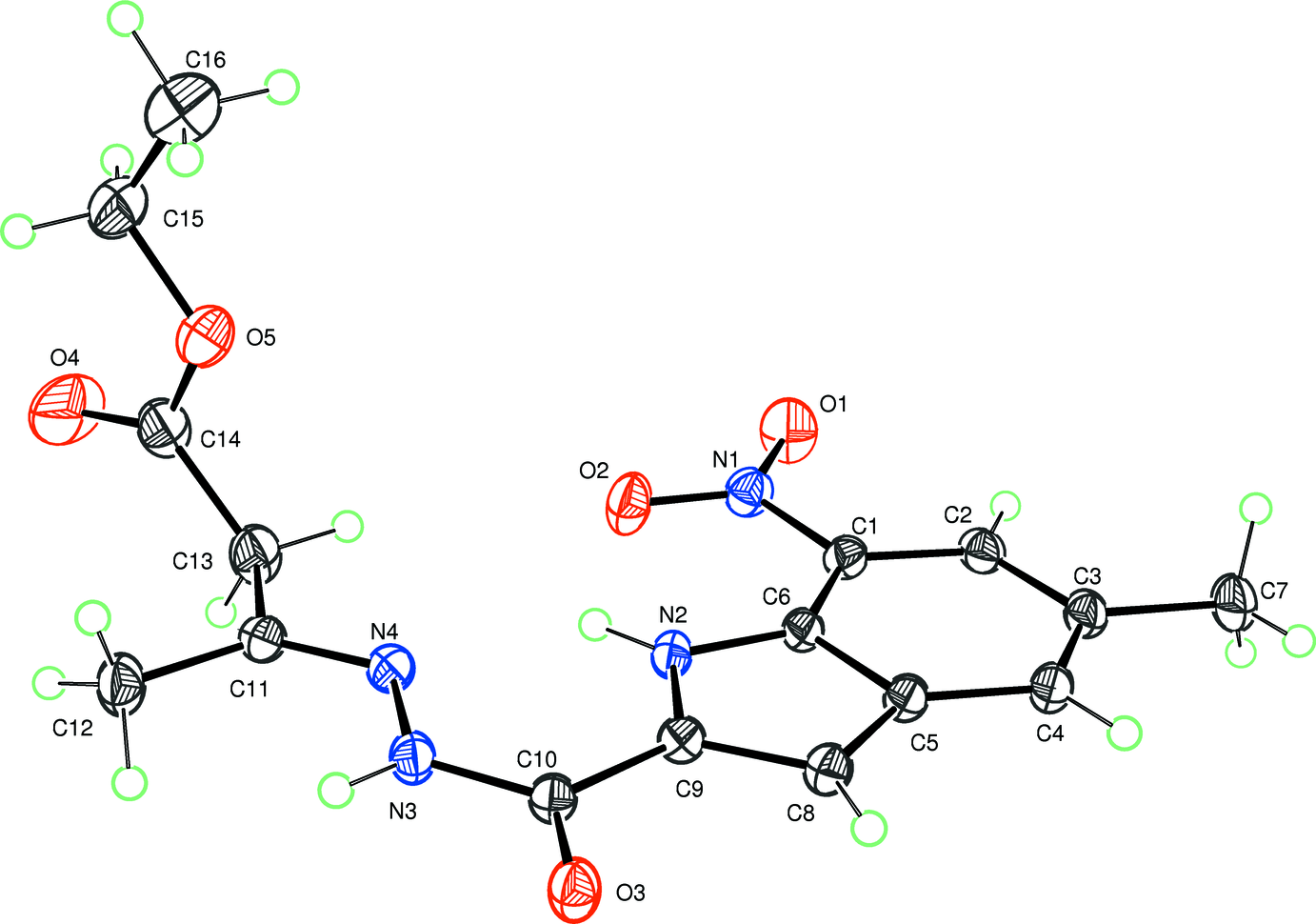

Supplement: Supplementary file 4 [file e-71-0o684-fig1.tif]

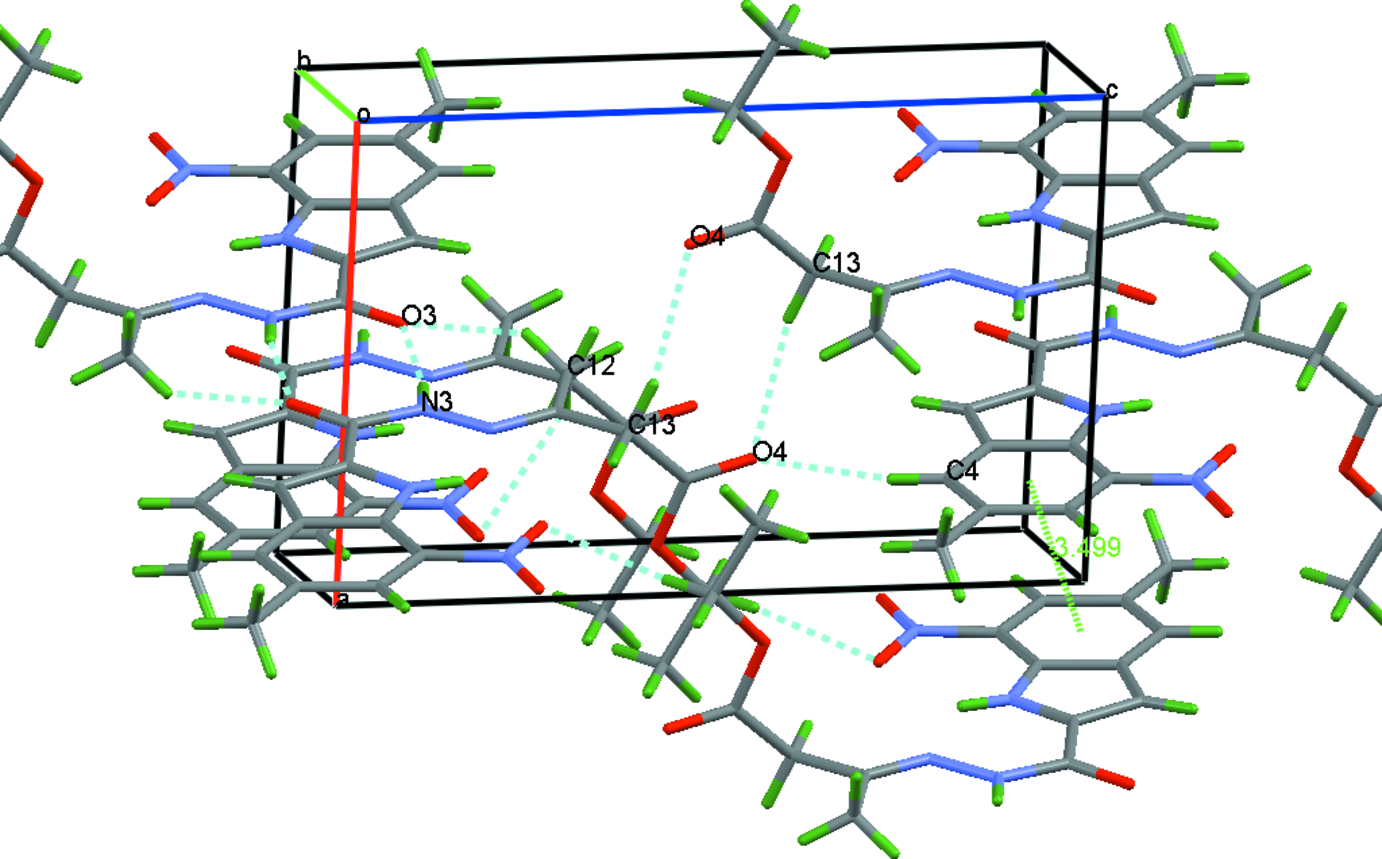

Supplement: Supplementary file 5 [file e-71-0o684-fig2.tif]
